# Metabolic Regulation of Hematopoietic Stem Cells

**DOI:** 10.1097/HS9.0000000000000740

**Published:** 2022-06-28

**Authors:** Claudia Morganti, Nina Cabezas-Wallscheid, Keisuke Ito

**Affiliations:** 1 Ruth L. and David S. Gottesman Institute for Stem Cell and Regenerative Medicine Research, Albert Einstein College of Medicine, Bronx, NY, USA; 2 Departments of Cell Biology and Stem Cell Institute, Albert Einstein College of Medicine, Bronx, NY, USA; 3 Department of Medicine, Montefiore Medical Center, Albert Einstein College of Medicine, Bronx, NY, USA; 4 Max Planck Institute of Immunobiology and Epigenetics, Freiburg, Germany; 5 Albert Einstein Cancer Center and Diabetes Research Center, Albert Einstein College of Medicine, Bronx, NY, USA

## Abstract

Cellular metabolism is a key regulator of hematopoietic stem cell (HSC) maintenance. HSCs rely on anaerobic glycolysis for energy production to minimize the production of reactive oxygen species and shift toward mitochondrial oxidative phosphorylation upon differentiation. However, increasing evidence has shown that HSCs still maintain a certain level of mitochondrial activity in quiescence, and exhibit high mitochondrial membrane potential, which both support proper HSC function. Since glycolysis and the tricarboxylic acid (TCA) cycle are not directly connected in HSCs, other nutrient pathways, such as amino acid and fatty acid metabolism, generate acetyl-CoA and provide it to the TCA cycle. In this review, we discuss recent insights into the regulatory roles of cellular metabolism in HSCs. Understanding the metabolic requirements of healthy HSCs is of critical importance to the development of new therapies for hematological disorders.

## INTRODUCTION

**Figure 1.**
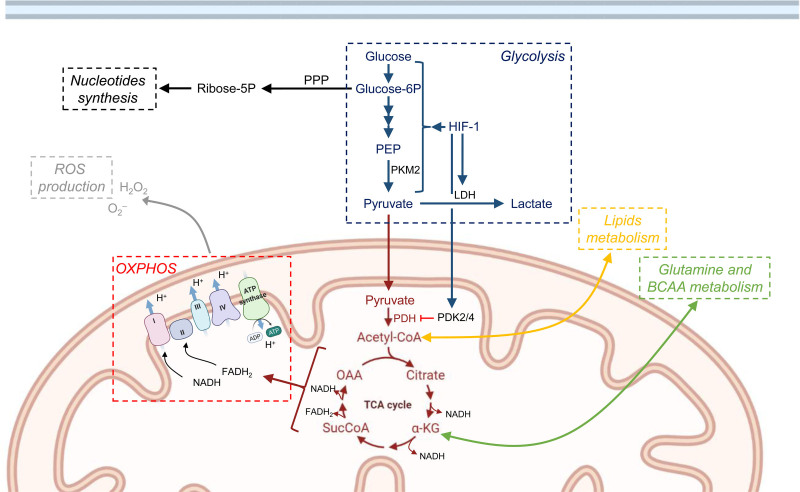
**Mitochondria play a central role in metabolic pathways contributing to HSC maintenance.** Overview of metabolic pathways contributing to HSC self-renewal and differentiation. HSCs mainly rely on glycolysis, which starts with the conversion of glucose into glucose-6P, and results in the generation of pyruvate, transformed from PEP by PKM2. Upon differentiation, pyruvate enters the TCA cycle in the mitochondria, fueling OXPHOS, and generating NADH and FADH_2_ to sustain high energy demands. OXPHOS produce ROS, and increased ROS negatively affects stem cell properties. To avoid the activation of OXPHOS and ROS production, HSCs utilize several alternative metabolism pathways. HIF-1 promotes glycolysis pathway and the conversion of pyruvate into lactate, enhancing the activity of LDH. Glucose-6P can be used by PPP to generate Ribose-5P to sustain nucleotides synthesis. HIF-1 also activates the PDK2/4, which is highly expressed in HSCs, and downregulates aerobic metabolism through inhibition of PDH-mediated conversion of pyruvate to acetyl-CoA. TCA cycle intermediates, Acetyl-CoA, and αKG link to lipids and amino acid metabolisms, respectively. Templates created with BioRender.com. -5P = -5 phosphate; -6P = 6-phosphate; HIF-1 = Hypoxia-inducible factor 1; HSC = hematopoietic stem cell; LDH = lactate dehydrogenase; OXPHOS = oxidative phosphorylation; PDH = pyruvate dehydrogenase; PDK = pyruvate dehydrogenase kinase; PEP = phosphoenolpyruvic acid; PKM2 = pyruvate kinase M2; PPP = pentose phosphate pathway; ROS = reactive oxygen species; TCA = tricarboxylic acid; αKG = α-ketoglutarate.

Hematopoiesis is defined as the formation of the cellular components of the blood. This cell production system endures for a lifetime thanks to the presence of hematopoietic stem cells (HSCs), which can give rise to additional stem cells by self-renewal or to committed cells through their multipotent differentiation capacity.^1,2^ However, over most of their lifetimes, HSCs remain in G_0_ of the cell cycle, that is, in a quiescent state, and undergo cell divisions only when required. The exit from quiescence and consequent differentiation of HSCs is synchronized with a metabolic switch from anaerobic glycolysis toward mitochondrial oxidative phosphorylation (OXPHOS).^3^ Mitochondria are biosynthetic hubs containing the major enzymes that oxidize carbohydrates, proteins, and lipids to produce adenosine triphosphate (ATP). Mitochondria fuel cellular metabolism through the production of ATP, a process that begins with the tricarboxylic acid (TCA) cycle housed in the mitochondrial matrix.^4^ Subsequent enzymatic steps of the TCA cycle result in the formation of reduced cofactors NADH and FADH_2_, which shuttle electrons to the electron transport chain (ETC) on the inner mitochondria membrane (IMM). The electron transfer of these electrons from NADH to oxygen allows protons to be pumped out of the matrix, which creates an electrochemical proton gradient in the IMM. The ETC ends at complex V (or ATP synthase), where the electrochemical proton gradient makes possible the conversion of ADP +Pi to ATP (OXPHOS).^5^ In addition to energy production, the mitochondria are key platforms for vital cell signaling cascade processes such as the regulation of reactive oxygen species (ROS) levels, calcium signaling, apoptosis, proteostasis, and heme synthesis.^6^ Cellular metabolism in HSCs has recently become an area of intense research interest^7,8^ and has been proposed as a key regulator of HSC maintenance.^9–12^ In this review, we discuss recent insights into the regulatory roles played by cellular metabolism in HSCs, with a particular focus on mitochondrial function. Beyond their central roles in energy production, mitochondria also participate in fatty acid metabolism and the biosynthesis of nucleotides and amino acids, and the roles played by these processes in HSC regulation are under continuing investigation (Figure 1).

## ANAEROBIC GLYCOLYSIS IS CRITICAL TO PRESERVE HSC QUIESCENCE

HSCs reside in the bone marrow niche, a hypoxic microenvironment that compels HSCs to rely on anaerobic glycolysis for energy production.^13,14^ Direct in vivo measurements of local oxygen tension (*p*O_2_) by 2-photon phosphorescence lifetime microscopy have determined the absolute *p*O_2_ of the bone marrow as <32 mm Hg, and have pinpointed the precise locations of HSCs and their niche with micrometer spatial resolution.^13^ Hypoxia-inducible factor (Hif)-1 and Hif-2 are key mediators of cellular responses to hypoxia. Although some studies with genetic mouse models showed that *Hif-1α* and *Hif-2α* as dispensable for HSC self-renewal,^15,16^ HSCs are also known to express high levels of Hif-1α.^14,17^ The deletion of *Meis Homeobox 1* (*Meis1*), a transcriptional activator of *Hif-1*, results in a shift to mitochondrial metabolism, and consequent increases in ROS production, which lead to loss of HSC quiescence and failure of bone marrow repopulation after transplantation.^18^ Exposure of HSCs to atmospheric oxygen likewise increases ROS levels in HSCs, which depletes HSC numbers and impairs their function.^19^ This evidence highlights the critical roles of the hypoxic microenvironment in preserving HSC quiescence.

HSCs show high expression of pyruvate dehydrogenase kinase (Pdk), a glycolytic enzyme which downregulates aerobic metabolism through inhibition of the pyruvate dehydrogenase (PDH)-mediated conversion of pyruvate to acetyl-CoA^20^ (Figure 1). Thus, elevated Pdk expression in HSCs leads to active suppression of the influx of glycolytic metabolites into the mitochondria. The effects of genetic deletion of *Pdk2* and *Pdk4* suggest that glycolytic metabolic status is a cell cycle checkpoint that modulates HSC quiescence and stem cell potential.^20^ Similar deletions of *Pyruvate kinase M2* (*Pkm2*), and *Lactate dehydrogenase A* (*Ldha*) have been shown to increase pyruvate entry into the TCA cycle (Figure 1), increasing oxidative phosphorylation and reducing HSC’ reconstitution potential in transplantation assays.^21^ Interestingly, mitochondrial activity and ROS production increased in HSCs only upon *Ldha* deficiency but not *Pkm2* deficiency, suggesting a complex regulatory network connecting anaerobic and aerobic metabolisms in HSCs.^21^ Treatment with an antioxidant can rescue the loss of function caused by deletion of *Ldha*,^21^ underlining the important role of mitochondrial ROS in HSC dysfunction.

Nevertheless, the glucose uptake rate is lower in HSCs than progenitors or more differentiated hematopoietic cells in the bone marrow.^22^ Liang et al showed that repression of lysosomal degradation by pharmacological V-ATPase inhibition promoted HSCs’ quiescence, which is associated with less glucose uptake. This led to improve ex vivo maintenance of functional HSCs and enhance their competitive repopulation ability upon transplantation.^23^ The higher glucose intake in active HSCs is required to sustain the entering the cell cycle, whereas quiescent HSCs maintain an overall low metabolic activity, which corresponds to a quiescent state. These findings imply that active HSCs require more energy. This state of metabolic quiescence preserves HSCs from premature exhaustion by cell proliferation and guarantees the long-term maintenance of HSCs.^23^

## STRATEGIES TO MINIMIZE ROS LEVELS IN HSCs

**Figure 2.**
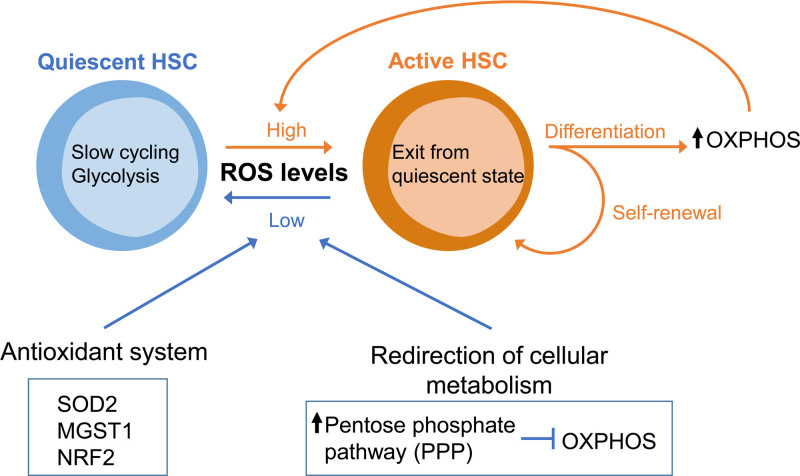
**Metabolic ROS control is necessary to preserve HSC quiescence.** Quiescent HSCs depend on glycolysis and exhibit low ROS level, whereas active HSCs instead increase proliferation and differentiation while switching to OXPHOS metabolism with high ROS production. ROS levels heavily influences HSC fate, thus antioxidant enzyme [SOD2, Mgst1, and NRF2] and redirection of cellular metabolism (fueling glucose metabolites into the PPP, instead of OXPHOS) maintain low the level of ROS in quiescent HSCs. HSC = hematopoietic stem cell; Mgst1 = microsomal glutathione transferase 1; NRF2 = nuclear factor erythroid 2-related factor 2; OXPHOS = oxidative phosphorylation; PPP = pentose phosphate pathway; ROS = reactive oxygen species ; SOD2 = superoxide dismutase.

HSCs exhibit low ROS levels compared with differentiated hematopoietic cells,^3^ and HSCs with lower levels of ROS retain higher stem cell potential.^24–26^ Metabolic ROS accumulates when HSCs exit from quiescence and proliferate in reaction to various DNA damage-inducing stresses.^27^ A number of genetic mouse models with increased ROS levels (eg, deletion of *Ataxia telangiectasia mutated*,^24,28^ *Tuberous sclerosis complex subunit 1*,^29^ and *Forkhead box O* (*FoxO*) family members^30,31^) have shown similar reductions in HSC function. HSCs have several strategies for regulating the production of ROS, including antioxidant enzymes and the shutdown of OXPHOS activation (Figure 2). The major antioxidant enzyme is superoxide dismutase (SOD). When SOD2 is reduced due to deletion of *FoxO3*, ROS accumulate in HSCs, leading to loss of HSC quiescence and exhaustion.^30,32^ Deficiency of *Microsomal glutathione transferase 1* (*Mgst1*), a glutathione-dependent enzyme which catalyzes biological redox reactions, results in defective HSC differentiation.^33^ Studies of the nuclear factor erythroid 2-related factor 2 (NRF2), a regulator of multiple antioxidant processes, have revealed that *Nrf2* knockout (KO) HSCs lose quiescence and stem cell potential.^34^ However, since antioxidant treatment with N-acetyl cysteine (NAC) cannot restore stem cell phenotypes, the ROS-independent roles of Nrf2 in HSC regulation should be further considered.^35^

Mitochondrial OXPHOS is the major source of cellular ROS production. Specifically, in mitochondria isolated from most tissues and incubated under physiological conditions, 0.1–0.2% of oxygen consumed converts to ROS through the ETC.^36^ Shifting glucose metabolites into the pentose phosphate pathway (PPP) rather than the TCA cycle is another mechanism that reduces ROS formation.^37^ The activity of the key enzyme for PPP, glucose-6-phosphate dehydrogenase, is regulated by a cytoplasmic NAD^+^-dependent deacetylase, Sirtuin 2 (SIRT2).^38^ Interestingly, upon aging, reduced SIRT2 expression and increased mitochondrial stress lead to aberrant activation of the NLRP3 (NACHT, LRR, and PYD domains-containing protein 3) inflammasome in HSCs, which drives functional decline of HSCs.^39–41^ The ability to regulate ROS levels is also critical because ROS affects chromatin remodeling and dynamics to heavily influence HSC stem cell fate.^42^ For example, hydroxyl radicals stimulate the conversion of 5-methylcytosine (5mC) to 5-hydroxymethylcytosine (5hmC), which promotes aberrant transcriptional activation.^42^ The ten-eleven translocation (TET) protein family is a critical regulator of the oxidation of 5-mC to 5-hmC, and the role of TET2 in normal and malignant hematopoiesis has been intensely investigated.^43–46^

## MITOCHONDRIA ARE NOT INACTIVE IN Hscs

Although HSCs mainly depend on glycolysis for energy production and OXPHOS is limited to minimize ROS production, certain mitochondrial activity can be detected in HSCs. Increasing evidence suggests that the mitochondrial content and membrane potential in HSCs has previously been underestimated, and the regulation of mitochondrial physiology has been highlighted as one of the key cell-intrinsic biological signals for proper HSC maintenance.^47^

Mitochondrial membrane potential (ΔΨ_mt_) is a key parameter in the assessment of mitochondrial functionality. ΔΨ_mt_ results from the equilibrium of proton pumping activity in the ETC and the proton flow through F_1_/F_O_ ATP synthase.^48^ Taking advantage of the electronegativity of the mitochondrial compartment, potentiometric dyes, such as tetramethylrhodamine methyl ester perchlorate (TMRM) have been developed to measure ΔΨ_mt_. TMRM has been extensively used in flow cytometry in a variety of cells,^49^ including hematopoietic stem and progenitor cells.^50^ The intake of TMRM dye depends on the negative charge of mitochondria, which is in constant balance with its clearance due to efflux pumps activity.^51^ Notably, the higher expression of the xenobiotic efflux pump in HSCs than in mature cell populations^52^ can affect this balance leading to biased results. This mechanism has been widely utilized in studies and investigations. Indeed, the extrusion of mitochondrial dyes such as Rhodamine 123 has allowed researchers to isolate HSCs^53^ or identify HSC “side populations” by exploiting the differential extrusion of the dyes Hoechst Blue and Hoechst Red.^54,55^ However, after the publication of findings that Fumitremorgin C, a specific blocker of the ATP-binding cassette subfamily G member 2 (Abcg2) transporter, does not affect the staining pattern of MitoTracker in HSCs,^14^ multiple studies were performed using mitochondrial dyes in the absence of xenobiotic efflux pump inhibitors. This led to the widespread belief that HSCs have only a small number of mitochondria with low ΔΨ_mt_.^14,56,57^

**Figure 3.**
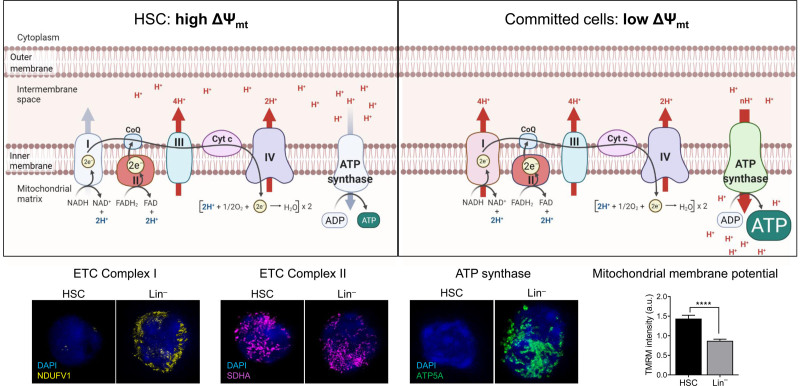
**The balance between electron transport chain complexes sustains high mitochondrial membrane potential in HSC.** Top: Schematic representation of the ETC complex in HSC and committed cells. ETC complexes I–IV transfer protons from the mitochondrial matrix to the periplasmic space to contribute to increase mitochondrial membrane potential (ΔΨ_mt_). This proton-motive force of ΔΨ_mt_ is used and depolarized by F_1_F_O_ ATP synthase (or complex V) to generate ATP. Unlike complex I and complex V, complex II expression is similar between HSCs and mature populations. This allows HSCs to sustain a high ΔΨ_mt_, which cannot be dissipated by ATP synthase, since it is barely expressed. Bottom: Representative immunofluorescence images of NDUFV1 (ETC complex I), SDHA (ETC complex II) and ATP5A (ATP Synthase) in HSC and committed cells (Lin^–^). Mitochondrial membrane potential measured by TMRM in HSCs and Lin^–^ cells. Data are modified from.^59^ Template created with BioRender.com. ATP = adenosine triphosphate; ETC = electron transport chain; HSC = hematopoietic stem cell; TMRM = tetramethylrhodamine methyl ester perchlorate.

This belief was challenged by the recent demonstration that Verapamil, a wide spectrum inhibitor of efflux pumps, significantly modifies the staining pattern of the mitochondrial dye MitoTracker Green.^58^ Indeed, Fumitremorgin C is highly selective for Abcg2, but is weakly selective for other transporters such as Abcb1a, which is highly expressed in HSCs.^58^ Similar results were obtained using both a Ca^2+^-independent multidrug resistance-mediated efflux inhibitor, Cyclosporin H, and other mitochondrial dyes, such as TMRM, Nonyl acridine orange, and Mitotracker Orange.^50,59^ Thanks to a dedicated protocol for accurate ΔΨ_mt_ measurement by TMRM-based flow cytometry, which corrects for xenobiotic transporter activity by proper inhibitors, such as Verapamil,^60^ it has been demonstrated that HSCs display the highest ΔΨ_mt_ among hematopoietic populations^59^ (Figure 3).

High ΔΨ_mt_ is not only meaningful to output and its measurement, but also supports proper HSC function; indeed, treatment with MitoQ, an antioxidant that localizes to mitochondria, inhibits the accumulation of mitochondrial ROS and increases ΔΨ_mt_, which enhances in vivo reconstitution potential upon transplantation of depolarized HSCs from aged mice.^61^ A similarly corrected approach to measuring mitochondrial mass by dyes has shown that HSCs exhibit similar or higher mitochondrial mass compared to other hematopoietic cells,^50,58,61^ a finding that has been repeatedly confirmed through 3D image-based content analysis of staining of mitochondrial structure antibodies (eg, TOM20).^50^

## ELECTRON TRANSPORT CHAIN IS CRITICAL FOR HSC MAINTENANCE

Despite high mitochondria content and high ΔΨ_mt_, mitochondrial ATP and ROS production is relatively inert in HSCs. This has raised questions regarding how mitochondrial activity is involved in HSC maintenance and proliferation, and how ΔΨ_mt_ is sustained at a high level in HSCs. Studies of the ETC in mitochondria have highlighted the importance of mitochondrial respiration for proliferation and HSC maintenance.^62,63^ Rieske iron-sulfur protein (RISP), an essential subunit of ETC complex III, is crucial for HSC quiescence and its loss leads to severe pancytopenia.^63^ Inducible *Succinate dehydrogenase complex subunit D* (*SdhD*)-ESR mutant mice exhibit induced deletion of the mitochondrial protein-encoding *SdhD* gene (which encodes a subunit of ETC complex II), which leads to decreases in long-term HSCs and committed progenitors of the myeloid lineage.^62^ We have further demonstrated that the expression and activity levels of ETC complex II relative to ETC complex V (or ATP synthase) are higher in hematopoietic stem and progenitor cells (HSPCs) than in more differentiated cells. Since the balance between proton pumping (by ETC complexes I-III) and proton flow (by ETC complex V) determines ΔΨ_mt_ levels, the higher ETC complex II: ETC complex V ratio in HSCs can explain their high ΔΨ_mt_ levels (Figure 3). Moreover, pharmacological inhibition of complex II by TTFA drastically reduces HSC ΔΨ_mt_ and in vitro long-term culture-initiating cell capacity.^59^ Additionally, ETC complex II links ETC and TCA cycle, but its precise roles or regulatory pathways in HSCs are still under investigation. Interestingly, it has been proposed that fumarate hydratase 1 (Fh1), another enzyme of the TCA cycle, which catalyzes the reaction from fumarate to malate, is involved in HSC maintenance, increasing the frequency of phenotypically defined HSCs, but compromising their self-renewal and differentiation.^64^ Similarly, the disruption of mitochondrial OXPHOS upon the loss of *Pten-like mitochondrial phosphatase* (*Ptpmt1*), a mitochondrial phosphatase targeting phosphatidyl inositol phosphates, blocks early HSC differentiation, which leads to rapid hematopoietic failure.^65^

Since glycolysis and TCA cycle are mainly disconnected in HSCs, other nutrient pathways, including amino acid and fatty acid metabolism, may generate acetyl-CoA and fuel the TCA cycle in HSCs and restricted progenitors.

## AMINO ACID METABOLISM AND NUTRIENT AVAILABILITY

Bone marrow contains 100-fold higher concentrations of all 20 amino acids than peripheral blood.^66^ However, little is known about the role of single amino acids in HSC function. Here, we provide 2 examples (glutamine and BCAA) of how amino acids contribute to HSC homeostasis.

**Figure 4.**
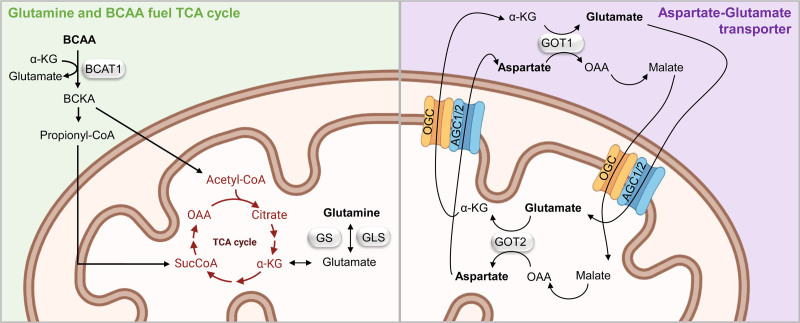
**Glutamine and BCAA fuel TCA cycle.** Green box: glutamine and BCAA pathway. In the mitochondria, glutamine is converted in glutamate by Gls and then αKG to fuel TCA cycle. Catabolism of BCAA by BCAT1 produces cytosolic Glu. The resulting BCKA fuel the TCA cycle via acetyl-CoA or are converted into propionyl-CoA, which enters the TCA cycle producing succinyl-COa (SucCoA). Purple box: glutamate-aspartate transporter. In the cytosol, OAA is converted into malate, which is then imported into the mitochondria by OCG in exchange for αKG. In the mitochondria, malate is reconverted in OAA, which in turn is transformed into aspartate by GOT2. GOT2 also produces αKG, which is used to exchange malate from the cytosol to the mitochondria. AGC1/2 simultaneously transports glutamate from the cytosol to the mitochondrial matrix, whereas exporting aspartate from the matrix to the cytosol. In the cytosol, OAA and glutamate are regenerated by GOT1. Silver box label enzymes. Template created with BioRender.com. AGC1/2 = Aspartate/glutamate carrier 1 and 2; BCAA = branched-chain amino acid; BCAT1 = BCAA transaminase 1; BCKA = branched chain keto acids; Gls = glutaminase; Glu = glutamate; GOT2 = glutamic-oxaloacetic transaminase 2; OAA = oxaloacetate; OCG = 2-oxoglutarate carrier; TCA = tricarboxylic acid; αKG = α-ketoglutarate.

Glutamine metabolism fuels the TCA cycle, since glutaminase (Gls) converts glutamine to glutamate and then to α-ketoglutarate (αKG) in the mitochondria (Figure 4). Alternative polyadenylation regulates the switching of Gls isoforms, resulting in upregulation of glutamine metabolism, which induces HSC transition from quiescence to proliferation.^67^ Moreover, glutamine metabolism affects HSC lineage commitment, since erythroid specification of HSCs requires glutamine-dependent de novo nucleotide biosynthesis.^68^

Catabolism of branched-chain amino acid (BCAA) also result in production of α-KG, Acetyl-CoA and Succinyl-CoA (or SucCoA) (Figure 4). Single amino acid–depleted culture media has revealed that HSC proliferation and maintenance in the mice depends on the branched-chain amino acid valine.^66^ Branched-chain aminotransferase 1 (BCAT1) catalyzes early steps of BCAA catabolism and has been proposed as a regulator of leukemic stem cells due to its influence on Hif-1α activity.^69^ BCCA metabolism has been shown to have a regulative role in proliferation rather than in quiescence. Notably, mutations in isocitrate dehydrogenase (IDH), which converts isocitrate into αKG, are also implicated in leukemogenesis. Indeed, mutations in *IDH* lead to aberrant HSC self-renewal and leukemogenesis in part through inhibition of TET2,^70,71^ whose activity depends on 2-hydroxyglutarate (2-HG), a product of αKG. HSC function is also known to be limited by aspartate, purine, and asparagine availability during hematopoietic regeneration.^72^ Specifically, aspartate synthesis increases upon HSC activation, and genetic models designed to increase aspartate levels, eighter through the deletion of glutamic-oxaloacetic transaminase 1 or the overexpression of the glutamate/aspartate transporter, increase HSC function^72^ (Figure 4).

**Figure 5.**
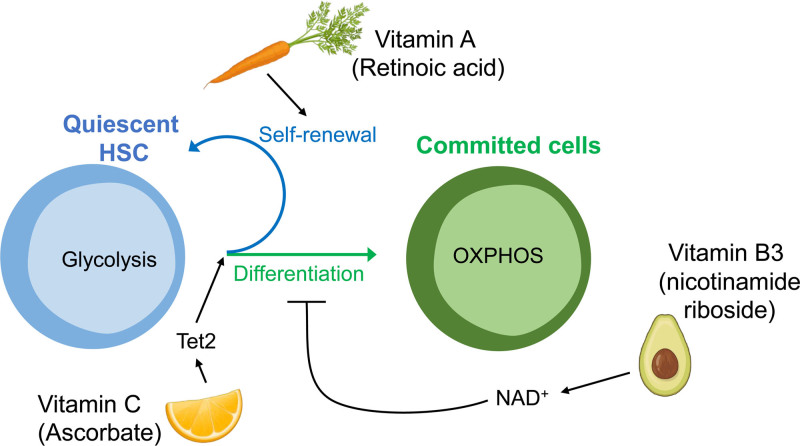
**Dietary habits maintain a healthy HSC pool.** Vitamin C (Ascorbate) is one of the most enriched metabolites in HSCs and decreased with differentiation. In homeostasis, Vitamin C regulates the balance between self-renewal and differentiation by promoting Tet2 activity. Vitamin A is crucial to maintain HSC quiescence, since Vitamin A-free diet leads to HSCs exhaustion and disrupted re-entry into dormancy. Dietary supplementation with the NAD^+^ precursor nicotinamide riboside, a form of vitamin B3, improves HSC function reducing mitochondrial metabolism. Icons created with BioRender.com. HSC = hematopoietic stem cell.

Not only amino acids, but also other metabolic cues are emerging as regulators of HSC function. Among these, specific vitamins (vitamin C, A, and B3, described below) are key regulators of transformed as well as normal HSCs, highlighting the relevance of dietary habits to maintaining a healthy stem cell pool (Figure 5).

Ascorbate (vitamin C) is one of the most enriched metabolites in HSCs, and decreases with differentiation.^73^ Ascorbate accumulates within HSCs to promote TET activity to limit HSC frequency and to suppress leukemogenesis.^73^ Since ascorbate treatment mimics Tet2 restoration by promoting DNA demethylation and reversing aberrant HSPC self-renewal, it has been suggested as a potential nontoxic therapy for TET-associated malignancies.^74^

Moreover, metabolites from the retinoic acid pathway (vitamin A) are also involved in the *in vivo* modulation of stem cell features. A vitamin A-free diet leads to HSCs exhaustion and disrupted re-entry into dormancy after exposure to inflammatory stress stimuli.^75^ Interestingly, a recent study has uncovered a nonclassical retinoic acid signaling axis that regulates HSC function, highlighting the idea that a single metabolite can control stem cell fate through epigenetic and transcriptional regulation.^76^ The low frequency of HSCs within the bone marrow has presented technical limitations for the study of specific modes of metabolism in HSCs. However, recent technical advances in low input metabolomics, combined with epigenetic/transcriptomic tools such as ATAC-seq, ChIP-seq, and RNA-seq, have now enabled to further address the distinct metabolic features of HSCs and their immediate downstream progenitors.^73,77^

NAD^+^ is an essential coenzyme in redox reactions catalyzed by dehydrogenases including glycolysis and TCA cycle enzymes. Dietary supplementation with the NAD^+^ precursor nicotinamide riboside, a form of vitamin B3, improves HSC function in mice by increasing mitophagy (described below) and reducing mitochondrial metabolism.^78^ Dietary supplementation with NAD^+^ precursors also improves the function of aging stem cells in the hematopoietic system reducing mitochondrial stress, mass and network-size.^79^

## LIPID METABOLISM

**Figure 6.**
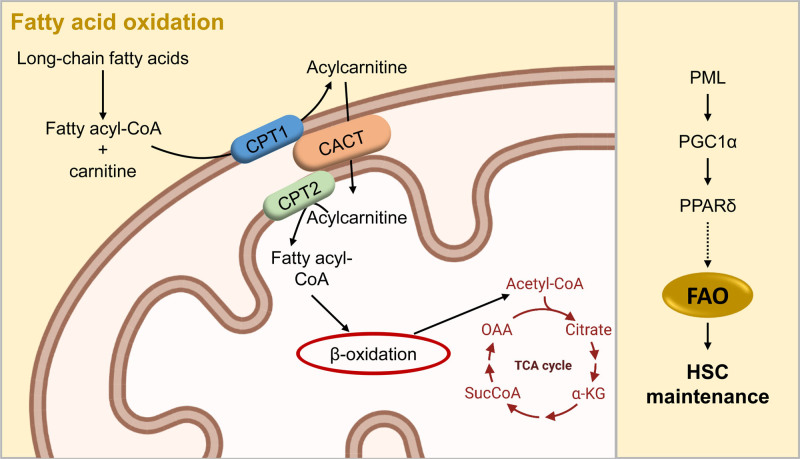
**Fatty acid oxidation sustains HSCs.** FAO, a key catabolic pathway for energy production, fuels the TCA cycle rather than glycolysis. Long-chain fatty acids are first activated in the cytosol in fatty acyl-CoA, and then transported by the carnitine shuttle system, which is composed by CPT1, CACT, and CPT2, into the mitochondria. Here, β-oxidation through multi-step reactions generates acetyl-CoA, which fuels the TCA cycle. Mitochondrial FAO is critical for HSC maintenance: PML regulates the PPARδ, through the transcription factor PGC1α. PPARδ is a regulator of FAO, and its deletion results in loss of HSC reconstitution potential. Template created with BioRender.com. CACT = carnitine-acylcarnitine translocase; CPT1 = carnitine palmitoyltransferase 1; CPT2 = carnitine palmitoyltransferase 2; FAO = fatty acid oxidation; HSC = hematopoietic stem cell; PGC1α = PPARg coactivator-1α; PML = promyelocytic leukemia; PPARδ = peroxisome-proliferator activated receptor delta; TCA = tricarboxylic acid.

Lipid metabolism, primarily fatty acid oxidation (FAO), participates in the regulation of HSC self-renewal and differentiation,^80,81^ and recent integrated–omics assays have shown that FAO is upregulated in HSC populations.^76^ In FAO, which is a key catabolic pathway for energy production, long-chain fatty acids are first activated in the cytosol, and then transported by the carnitine shuttle system into the mitochondria, where they undergo multistep reactions to generate acetyl-CoA (Figure 6). Acetyl-CoA enters the TCA cycle, whereas the reduced electron carriers NADH and FADH_2_ deliver electrons to the ETC.^82^ Our group has demonstrated that HSC maintenance depends on the mitochondrial FAO pathway. Indeed, the deletion of peroxisome-proliferator activated receptor (Ppar) delta, a regulator of fatty acid transport and oxidization, which is regulated by promyelocytic leukemia (Pml), results in loss of HSC reconstitution potential^80,83^ (Figure 6). The activation of the PPAR-FAO pathway promotes expansion of HSCs through the quality control of mitochondria by enhanced Parkin recruitment in the mitochondria and mitophagy.^84^ Interestingly, inhibition of Ppar gamma promotes ex vivo expansion of human cord blood hematopoietic stem and progenitor cells, causing a metabolic shift to glycolysis.^85^ More recently, it has been demonstrated that timely degradation of proteins by chaperone-mediated autophagy is required for upregulation of fatty acid metabolism upon HSC activation.^86^

Finally, there is increasing interest in lipolysis mechanism in bone marrow adipocytes (BMA), which are a critical competent of the bone marrow niche. BMA numbers are believed to correlate negatively with HSC function, since HSC numbers are reduced in the adipocyte-rich vertebrae of the mouse-tail compared to the adipocyte-free vertebrae of the thorax.^87^ Furthermore, pharmacological and genetic approaches to reducing adipogenesis promote engraftment following irradiation.^87^ However, BMA numbers were also reported to increase after irradiation concomitantly with HSC proliferation.^88^ BMA, together with leptin receptor-cre^+^ mesenchymal stem cells, were recently identified as the major sources of stem cell factor (Scf) after irradiation, and thus are essential for hematopoietic recovery.^89^ Quiescent HSCs are metabolically plastic and undergo minute changes and adaptations to the environment to meet metabolic demands, and recent studies have shown that to meet the metabolic needs of infection response, HSPCs uptake free fatty acids from their microenvironment to undergo a metabolic shift toward fatty acid metabolism.^90^ Future studies are needed to address these controversial results. However, it is reasonable to hypothesize that BMA may be essential for emergency hematopoiesis, yet may still be deleterious to this process when present in unbalanced numbers.^91^

## MITOCHONDRIAL DYNAMICS

**Figure 7.**
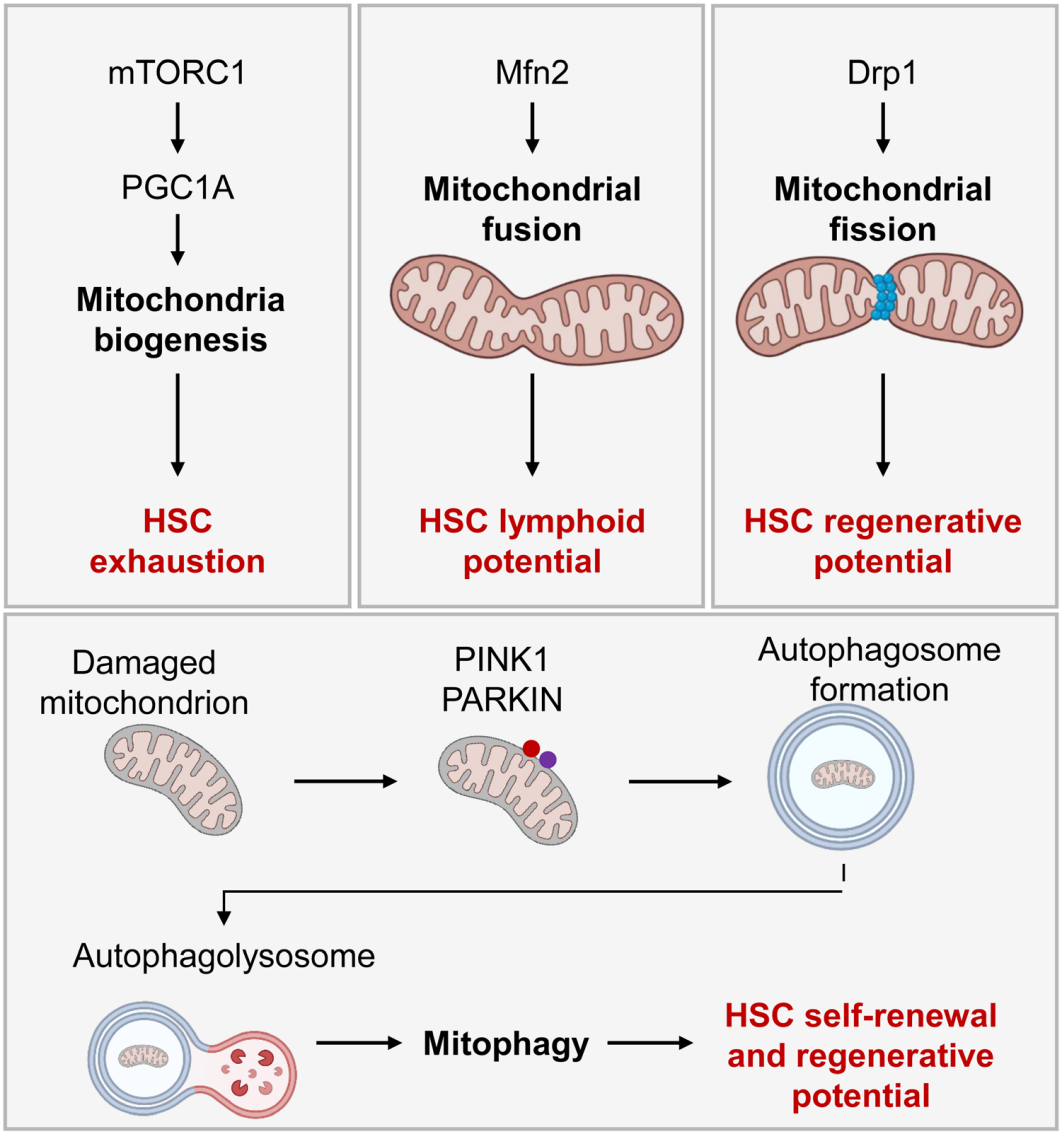
**Effects of mitochondria dynamism on HSC.** Mitochondrial biogenesis, fission, fusion, and mitophagy control mitochondrial mass and shape affecting HSC homeostasis. mTORC1 upregulates the transcription factor Pgc1α to activate mitochondria biogenesis, which leads to HSC exhaustion. Mfn2 promotes mitochondrial fusion and is specifically required for the maintenance of lymphoid potential HSCs but not myeloid-dominant HSCs. Drp1 creates a ring-like structure able to divide mitochondrial network filaments (mitochondrial fission). Deficiency for Drp1 causes loss of HSC regenerative potential while maintaining HSC quiescence. Damaged mitochondria are selectively cleared through mitophagy. Pink1 and ubiquitin ligase Parkin label damaged mitochondria, which are incorporated into the autophagosome and removed by fusion with the lysosome (authophagolysosome). Parkin recruitment in the mitochondria enhances HSC self-renewal, and lysosome activity is critical to maintaining HSC repopulation capacity. Icons created with BioRender.com. Drp1 = dynamin-related protein 1; HSC = hematopoietic stem cell; Mfn2 = mitofusion 2; mTORC1 = mammalian target of rapamycin complex-1; Pink1 = Pten-induced putative kinase 1.

Mitochondria are central players in cellular metabolism; therefore, fine-tuned mitochondrial dynamics are necessary for optimal response to cellular energy demands. Mitochondria mass and shape alter with mitochondria biogenesis, as well as fission, fusion, and mitophagy processes^92^ (Figure 7). Mammalian targets of rapamycin complex-1 (mTORC1) is involved in multiple cellular metabolism pathways, including mitochondrial biogenesis by upregulation of the transcription factor PPARg coactivator-1a (Pgc-1a).^93^ mTORC1 activation causes HSC exhaustion, whereas the deletion of Raptor, a component of mTORC1, results in pancytopenia and inhibition of HSC regeneration.^94^ However, the deletion of *Pgc1a* impairs the long-term reconstitution potential of HSCs in bone marrow transplantation with minimal effects on physiological HSCs.^95^

Mitochondria dynamically alter their shape though fission and fusion processes.^96^ Although mitochondrial fission and fusion are dispensable for HSC maintenance, they are critical for normal hematopoietic differentiation. Mitofusion 2 (Mfn2), which promotes mitochondrial fusion, is specifically required for the maintenance of lymphoid potential HSCs but not for myeloid-dominant HSCs.^97^ Dynamin-related protein 1 (Drp1) is recruited to mitochondria in order to create a ring-like structure able to divide mitochondrial network filaments in a process called mitochondrial fission.^98^ *Drp1* deficiency causes loss of HSC regenerative potential but maintains HSC quiescence. Due to the asymmetric segregation of aggregated mitochondria among hematopoietic progenitors during cell division, HSCs carrying dysfunctional mitochondria can re-enter quiescence, but fail in cell cycle regulation in subsequent division. Thus, loss of fidelity of mitochondrial control mechanisms drive HSC exhaustion.^99^

Mitophagy is the specific form of autophagy responsible for the selective clearance of damaged mitochondria, and the mitochondrial kinase Pten-induced putative kinase 1 (Pink1) and ubiquitin ligase Parkin play critical roles in this process.^100^ Our group has demonstrated that Parkin recruitment in the mitochondria enhances HSC expansion, and conversely, inhibition of mitophagy by acute silencing of Parkin limits HSC self-renewal.^84^ In asymmetric division, old and defective mitochondria can be segregated in 1 daughter cell in order to maintaining stemness properties,^101^ but in the case of symmetric self-renewing division, damaged organelles are inherited by both daughter cells.^102^ Thus a quality control process such as mitophagy is critical to controlling the level of defective organelles, which otherwise could represent a limiting factor on self-renewing division, and consequently HSC expansion. Enhanced mitophagy also affects HSCs. Accumulation of Pink1 caused by loss of AAA+-ATPase (Atad3a) hyperactivates mitophagy in HSCs.^103^ Deletion of *Pink1* in *Atad3a*-deficient mice has been shown to correct mitophagy defects and restore the progenitor and HSC pools.^103^ Lysosome activity is critical to completing the clearance of mitochondria,^104^ and the number of lysosome is high in quiescent HSCs and their activity preserves HSC features.^23^ The suppression of lysosomal acidification leads to lysosomal enlargement and increased mitochondria sequestration, which results in enhanced competitive repopulation ability.^23^

## CONCLUSION AND PERSPECTIVE

Over the past decade, an abundance of new genetic models and -omics techniques have expanded our knowledge of metabolic functions in adult HSCs, and it has become clear that metabolites play an active role in regulating cell fate. HSCs depend mainly on anaerobic glycolysis for energy production, and limit OXPHOS and ROS production in order to maintain quiescence and an undifferentiated state. Nevertheless, HSCs still maintain mitochondrial activity, and disruption of mitochondrial respiration is detrimental to HSC homeostasis. Since glycolysis and TCA cycle are not directly connected in HSCs, other nutrient pathways, specifically amino acid and fatty acid metabolism, generate acetyl-CoA and fuel the TCA cycle in HSCs and restricted progenitors. Recent work has further shown a link between acetyl-CoA metabolism and epigenetic regulation, particularly histone acetylation, in HSCs during hematopoietic recovery after myeloablation.^105^ The reliable measurement of single-cell metabolomics could define molecular profiling of HSCs during exit from quiescence and entrance into the differentiation process.^106^ Moreover, newly established techniques, such as the joint analysis of global data on gene expression, chromatin accessibility, and histone modifications, have revealed new metabolic players in HSC biology.^76^

Elucidating the mechanisms of HSC fate control is a key goal of ongoing research in HSC-based therapies, including long-term ex vivo HSC expansion and leukemia treatment. The analysis of different environmental factors allows improving HSC ex vivo expansion, highlighting the critical role for fatty acids.^107,108^ Recent studies have highlighted that leukemic stem cells (LSCs) and treatment-resistant cells are dependent on mitochondrial metabolism; thus, energy metabolism in LSCs is now considered a promising therapeutic target.^109^ For instance, the venetoclax/azacitidine effect on the eradication of acute myeloid LSCs depends on inhibition of amino acid metabolism,^110^ but resistance to venetoclax/azacitidine occurs via upregulation of FAO.^111^ Thereby, pharmacological inhibition of FAO restores sensitivity to venetoclax/azacitidine in drug resistant LSCs.^111^ Moreover, it has been shown that FAO is fueled by adipose tissue present in the microenvironment to sustain LSCs.^112^ Targeted mitochondria drugs, such as Actinomycin D, are critical to leukemia therapies,^113^ and defective mitochondria are found both in aging and preleukemic conditions.^40,41,114^ In conclusion, advances in our understanding of the pivotal metabolic requirements of healthy HSCs are critical to developing new therapies for hematological disorders.

## ACKNOWLEDGMENTS

We are grateful to members of the Ito lab and the Einstein Stem Cell Institute for their comments on the topics of hematology and stem cell biology.

## AUTHORS’ CONTRIBUTIONS

CM wrote the article. NCW and KI edited the article. CM designed the figures.

## DISCLOSURES

The authors have no conflicts of interest to disclose.

## SOURCES OF FUNDING

KI is supported by grants from the National Institutes of Health (R01HL148852, R01DK098263, R01HL069438, and R01DK115577). KI is a Research Scholar of the Leukemia & Lymphoma Society (no. 1360-19). NC-W is supported by Max Planck Society and the ERC-Stg-2017 (VitASTEM). CM is supported by The Einstein Training Program in Stem Cell Research, which is acknowledged from the Empire State Stem Cell Fund through New York State Department of Health Contract (C34874GG).
